# Dental Education

**Published:** 1871-05

**Authors:** H. Scott


### DENTAL EDUCATION.

BY H. SCOTT.

It is a matter of pride to every dentist who loves his profession, to know that so much is being done to enlarge ard popularize dental science. The efforts to this end are seen in colleges for the special preparation of the mind and hand for professional duty; in the extent and ability of dental literature; and in the formation of national, state, and district co-operative and mutual aid societies. In the van of all these movements are men of high mental and educational ability. We have also with us a large amount of inventive genius. The plan of education for dental colleges is broad. A man can not graduate in any of our institutions of learning, with less than a respectable acquaintance with anatomy, physiology, chemistry, pharmacy and therapeutics, in addition to a thorough knowledge of all the specialties of dental theory and practice, besides being a respectable English scholar. He must also show in the laboratory a respectably constructive and manipulative ability, and with all, fill the measure, pretty nearly, of a gentleman in his sphere of life. This is the high stand our colleges have taken, and is all well and right. With every meeting of societies, as also with the monthly and quarterly appearance of our periodicals, new lights are emited. Some new and better methods of doing things have been found by the pioneers of the profession, and with few exceptions, are contributed to the common stock. And thus, within little more than a quarter of a century, has the profession of dentistry come up from obscurity and almost contempt, to rank with the learned and popular institutions of the age. There is no movement of the nineteenth century that has equaled ours in the rapidity and value of discovery, and successful practical application to the comforts and requirements of man. These are the facts that stimulated and encouraged us all to still greater and more useful achievements. This is all well; but are we not delinquent in plans for the required

EDUCATION OF THE MASSES?

How this is to be done, is the question to be talked over. We might prepare and publish for the people such facts as they are most interested in understanding, and the knowing of which would render the dentists professional intercourse intellectual, and mutually agreeable, besides lightening his labors, and at the same time, increasing his pecuniary interests. The truth is, that with the exception of one or two in one or two thousand, the people know less of their teeth, physiologically, pathologically or remedially, than of any subject that concerns their welfare; and this is true of large numbers of both sexes who, upon almost every other question are well informed. How many can any operator find among his patrons, even the best educated and most intelligent of them, who can tell for their lives what the teeth are made of? Where will you find one who knows that every tooth has a nerve, and an artery, and a vein entering at the point of every root, through an opening sometimes too small to be seen with the natural eye, and all sheathed with an exceedingly delicate and sensitive membrane, and that these widening out in the crown, form the pulp or nerve of the tooth, as it is common to say. A very great many people, otherwise intelligent, believe to-day that every tooth has a worm in it, which causes toothache by biting, perhaps, or by wiggling its tail. We often hear people speak of the worm in their tooth. It is due to everybody that has teeth, that they should be instructed in the anatomy, physiology and pathology, as well as cures of the teeth; and also, that being connected with the general system by deriving their nerves from the same trunks that send branches to the ear, face, scalp and other parts, morbid conditions of them often induce severe pain in remote parts, by nervous sympathy. They should also know that inflammation of the investing membranes is sometimes the cause of the most intense pain.

Who that has been a dentist a few years has not been almost daily enlivened with the following and similar questions: "*Doctor, do you know anything that is good for aralgy?*" You examine the mouth and find the source of the "*aralgy.*" But that can't be possible, for "*the tooth has never ached,*" and before you can arrive at an understanding, and do what the case requires, you will have consumed as much time as you should consume in relieving two or half a dozen cases. And then, "*I don't think its toothache; I think I have taken cold.*" *And*, "*why doctor, how can a tooth ache when it has no nerve in it?*" One will insist that he never shed his first teeth, another that he has double teeth all round, and occasionally you find one that "*has his third set.*" Well, this is all excusable, for none of us know anything till we have learned it.

A liberal and general special education of the whole people, on all matters concerning the teeth and their cures, would also sooner and much more effectually rid society of incompetent and mere mercenary operators, than any laws can do. I rather doubt the ultimate success of laws regulating medical or dental practice. We shall see in time, but my observation has been, that a majority of the common or non-professional people, have been inclined to regard all such legislation as partial and intended to favor the few. I take it for granted that every body is aware that the people, as a rule, are impatient of legislation conferring special privileges on societies or parties. Of one thing I think I am sure, that there are as many quack dentists to-day as there were before the existing dental law was passed, and I know of no way on earth to make them less, other than starving them out. Dentists can not become informers, for that would be jealous persecution. The people will not present them, but they would let them alone if they could duly and intelligently understand that their personal interest would be served in doing so.

I have sometimes thought that a dentist who would keep in the straight and narrow way, should attend not less than two methodist camp meetings each year, if he would not fall from grace, or, on the other hand, that he might be converted over again as often as he lost his religion. It is less than a week since, that a lady called at my office with her two daughters, of 15 and 13 years respectively. I have had charge of this lady's teeth, as well as those of her husband, for more than twenty years, and could as easily have conceived of any event in the world transpiring, as that they would have taken their daughters to any office other than mine to have their teeth filled, or for any advice concerning them. I had a right to feel and to believe so, for they have always spoken of me as a friend, and have counciled their friends to come to me for professional services. What was my surprise and mortification, as well, on finding that the girls had been thirty miles from home attending school, and had found a dentist who assured them that amalgam was far better every way to fill teeth with than gold, and actually, with the approval of their father, had some incisors with small cavities filled with an imperfectly prepared amalgam, which was already of an inky color. I felt then, and so expressed myself to the mother, that self-respect would require me to withdraw from my profession in very shame, and never touch another tooth while I lived, but better thoughts have since prevailed. And there are many such trials of patience. I could write a volume about them. A lady, whose teeth I filled thirty years ago, sent her daughter recently to have two incisors filled. She would not allow the file used. Somebody had told her that the file would break the enamel.

I spent a reasonable length of time in trying to satisfy her that the ragged and crumbling borders must be trimmed back to ensure successful filling, but I could not overcome her prejudices, and was obliged to dismiss her, though she was above twenty years of age. I suppose every dentist has his share of such experiences. It must be to an educated people, at last, that we are to look for the final success of our profession; and let us see finally how such education can most effectually be secured.

Every educated dentist could write out the very lessons important to be learned, but how could they be printed and placed in the hands of every family? Newspapers will not, and should not publish such matter without pay, and who can afford to pay for so much printing out of his own pocket. But suppose such literature were distributed gratuitously, how is its study to be insured while Bonner's *Ledger*, the *New York Weekly*, and hundreds of such sheets, are sent off by the car load to fill every house in christendom with cheap reading. It would be almost a marvel to-day, if a single house could be discovered, where these frivolous, if not directly demoralizing sheets do not constitute the greater part, if not the entire family reading, while sensible books, including the sacred volume, are peacefully corroding away on the shelf. The infatuation for this fictitious and low literature is appalling, and disreputable to an enlightened people. Think of our women chiding their husbands and sons on account of their tobacco and lager, and then think of their minds being chained down to the low thoughts and caricatures of human nature, that make up about the sum total of the cheap literature of the day.

If any good is to be done in the way of elevating the public mind up to an intelligent understanding of the advantages of dental science, and its practical utility and application to the requirements of the mouth; I apprehend that it must be accomplished through the instrumentality of our common school system. As the world is now, men and women have no time to devote to useful study. The acquisition of money requires all the time of nearly every person, leaving just interval sufficient for sleep and the reading of the *Ledger*, *Weekly*, etc. Men and women ought to be educated in the art of taking care of their bodies, and of preserving the best physical health; for without sound organisms, there can scarcely be sound and vigorous minds. The public funds should pay for the public education, and this should include the study of the teeth and their diseases and remedies. In no other way does it seem possible that this end can be accomplished, and that the study may become a part of common school education. Dental associations should work to awaken the attention of legislatures.

